# Population Dynamics of a Two Phages–One Host Infection System Using *Escherichia coli* Strain ECOR57 and Phages vB_EcoP_SU10 and vB_EcoD_SU57

**DOI:** 10.3390/ph15030268

**Published:** 2022-02-22

**Authors:** Shazeeda Koonjan, Carlos Cardoso Palacios, Anders S. Nilsson

**Affiliations:** 1 Department of Molecular Biosciences, The Wenner-Gren Institute, Stockholm University, SE-106 91 Stockholm, Sweden; carlos.cardoso.palacios@slu.se; 2 Department of Ecology, Grimsö Wildlife Research Station, Swedish University of Agricultural Sciences, SE-739 93 Riddarhyttan, Sweden

**Keywords:** population dynamics, chemostat, evolution, coevolution, resistance, cross-resistance, phage therapy, diversity, microbiome

## Abstract

In this study, we looked at the population dynamics of a two phages-one host system using phages vB_EcoP_SU10 (SU10) and vB_EcoD_SU57 (SU57) and the bacteria *Escherichia coli*, strain ECOR57. Phage-specific growth curves were observed where infections by SU10 resulted in a moderate production of phages and infections by SU57 resulted in a fast and extensive production of phage progeny. Sequentially adding SU10 followed by SU57 did not produce a significant change in growth rates, whereas adding SU57 followed by SU10 resulted in a decrease in SU10 titer The efficiency of the plating assays showed that ECOR57 exhibited a resistance spectrum after infection by both the single and combined phages. Phage-resistant bacteria exhibited four different morphotypes (i.e., normal, slimy, edgy, and pointy). The normal and edgy morphotypes had a high frequency of developing resistance. Bacterial growth and biofilm assays indicated that the edgy and pointy morphotypes reached a stationary phase faster and produced more biofilm compared to the wild type. These findings suggest that the dynamic structure of phage–bacteria communities dictate resistance evolution and development. Understanding when and how resistances arise and phage(s)–hosts interactions could aid in the design of phage therapy treatments.

## 1. Introduction

A substantial fraction of the human microbiome is located within the gastrointestinal tract, which has a delicate balance that is maintained by a variety of microorganisms such as bacteria and viruses [1]. Abnormal changes to gut flora composition and concentration, otherwise known as dysbiosis, often occur following antibiotic treatments. Dysbiosis has been associated with diseases such as irritable bowel syndrome, inflammatory bowel diseases such as Crohn’s disease, and colorectal cancer [2]. 

Viruses infecting bacteria, also known as bacteriophages (phages), maintain gut bacteria diversification and homeostasis [3,4]. Composed of more than 10^10^ per gram of the gut microbiome, phages are species specific and can play a role in bacterial genetic exchange through transduction [3,5,6,7]. Phages can be either virulent or temperate and following infection, phages can undergo different replication cycles, the most common being lysogenic and lytic [8]. Infections by virulent phages, and most infections by temperate phages, result in bacterial lysis shortly after infection, but temperate phages can also either integrate into the host genome and become a prophage or be maintained in the bacterium as an episomal element. As the bacterial host undergoes replication, the phage genome is also replicated and passed down to bacterial progeny [9]. Phages are commonly found as such prophages in bacteria within the gut and, in some cases, can contribute to at least 20% of their bacterial host genome [10,11,12]. Often these prophages have a symbiotic relationship with their host bacteria, while temperate phages can introduce bacterial virulence factors or other fitness enhancing genes resulting in lysogenic conversion of bacteria [13]. Under stressful conditions, such as DNA damage, prophages and/or episomal elements can be induced to undergo lytic replication [14,15,16]. Virulent phages undergoing lytic replication, on the other hand, take over bacterial replication machinery to replicate its viral genome, produce viral structural proteins, and assemble phage progeny [14,17]. The lytic cycle culminates with bacterial lysis and the release of phage progeny [18]. 

Bacteria can reduce adsorption and phage infection by employing phenotypic resistance mechanisms to prevent phages binding to their cell surface receptors. Phenotypic resistance can be divided into three categories: induced where exposure to phage-lysed bacteria triggers a gene expression change in uninfected bacteria, intrinsic where a physiological state or gene expression conferring resistance exists in a subset of bacteria population prior to phage introduction, and dynamic where phage proteins released when bacterial lysis degrades or blocks bacterial receptors [19]. Examples of phenotypic resistance includes the production of decoys in the form of outer membrane vesicles to which phages can bind to instead of producing post-translational receptor modifications, such as glycosylation, regulating receptor expression, and changing receptors in such a way as to prevent phage binding [20,21,22,23,24]. Bacteria can also prevent phage adsorption by hiding their receptors such as within biofilms [20,25,26]. 

Bacteria have a high rate of developing mutations in their anti-phage defense mechanisms against single phages, which can rapidly spread throughout the bacterial population [27,28,29]. Due to the fitness costs associated with carrying many defense mechanisms, bacteria must weigh the trade-offs between carrying these resistance systems and the benefit of resisting phage infection. This must also apply to bacteria in the human gut. Given the natural abundance, continuous presence, and varying virulence of phages, it is highly unlikely that gut bacteria are only susceptible to infections by single-phage “species”. Pressure from multiple phage infections can lead bacteria to mutate to cross-resistance, where the resistance against one phage results in resistance to other phages [28]. Intrinsically, bacteria can favor single mutations that affect common phage adsorption receptors such as outer membrane proteins, lipopolysaccharide, or pili, becoming resistant to all phages that use the same receptor [30]. Bacteria can also become cross resistant to phages that use different adsorption receptors by modulating common global regulators of multiple receptors [31,32]. The evolution of bacterial cross-resistance is driven by whether bacteria have been sequentially or simultaneously exposed to phages, the multiplicity of infection (MOI) of phages present for infection, as well as by the order each phage infects [28,33]. 

Employing anti-phage defense mechanisms, such as those previously described, are highly dependent on the type and infection kinetics of the infecting phage. Virulent phages ideally would have high adsorption rates, short latent periods, and large burst sizes [34]. However, due to the phage adaptation and bacterial population density, there has to be an evolutionary trade-off between latent periods and burst sizes [35,36,37]. Virulence could also be defined as those phages that have co-evolved with bacteria to overcome their anti-phage defense mechanisms [38,39]. Recently, the concept of “phage training” was introduced as a way of circumventing the evolution of phage resistance and increasing bacterial suppression [40,41]. However, it can be argued that phage training would cause a stronger selection on target bacteria and accelerate the evolution of resistance, which could be important to consider when applying phages against bacterial infections, while this may result in a reduction in therapeutic efficacy [41]. With the constant presence of phages in the gut microbiome, coinfection and phage interference by two or more virulent phages is bound to happen, which could result in synergism or the competition between phages for resources, the latter of the two being most probable [31,32,42]. This, in addition to phage dosing and reaching the site of infection, would have to be accounted for if phages are to be used therapeutically and successfully [43]. 

In this study, the population dynamics of a two phages–one host system was investigated. Strain 57 from the *Escherichia coli* reference collection [44] was infected with two unrelated, previously isolated and characterized lytic phages: *Podoviridae* C3 morphotype phage vB_EcoP_SU10 (SU10) and *Drexlerviridae* B1 morphotype phage vB_EcoD_SU57 (SU57) [35,45], in chemostat microenvironments. The population structure, size, and cell viability, in terms of resistance/susceptibility against those phages, were also investigated. Understanding the resistance development and population structure of a two phages–one host system can shed light on how the bacteria–phage equilibrium is maintained in the gut.

## 2. Results

### 2.1. Population Dynamics of a Two Phages–One Host System Using SU10/SU57 and ECOR57

**Figure 1:**
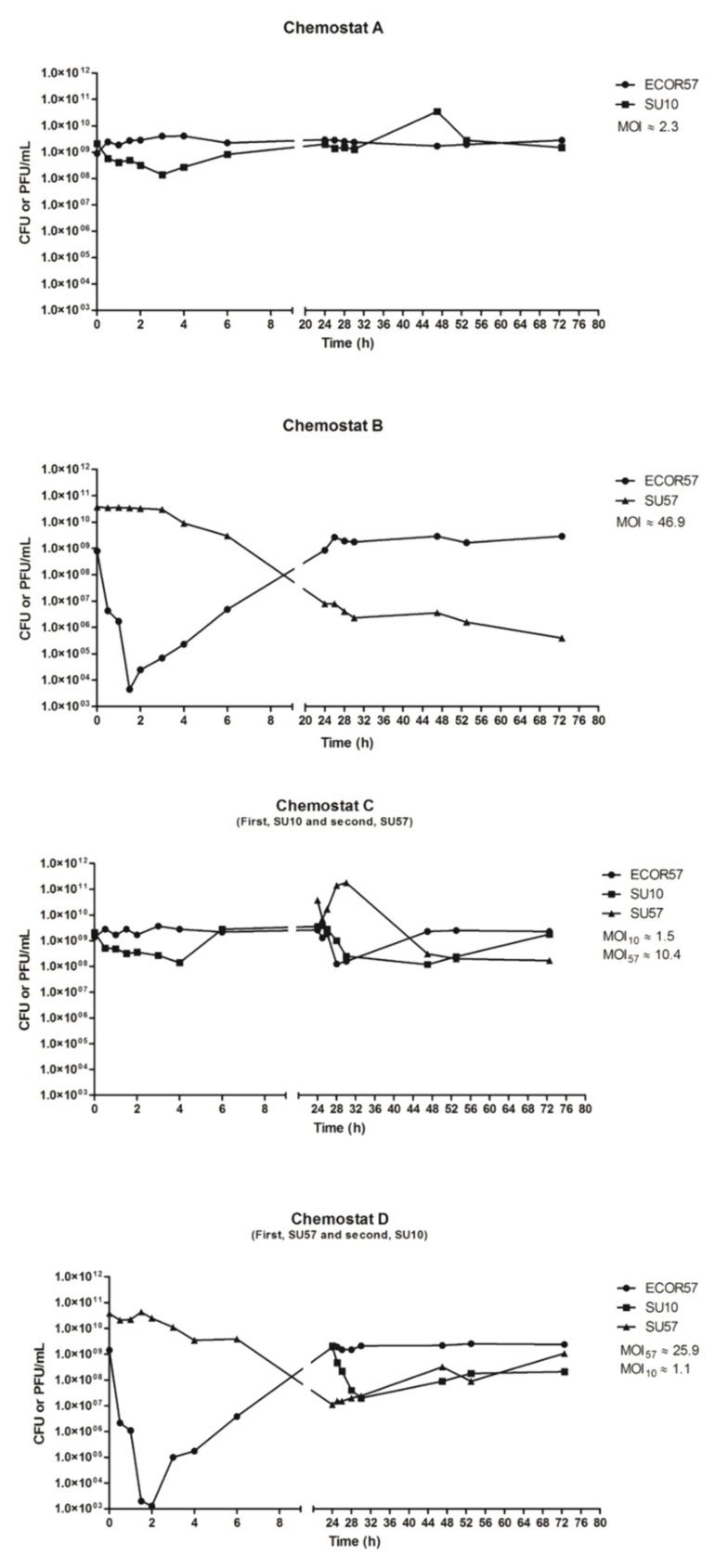
Population dynamics of ECOR57 under SU10 and/or SU57 phage treatments in chemostat microenvironments. Infection with SU10 and SU57 produced phage-specific characteristic population dynamic curves. (**A**) SU10 produced a moderate production of phages, where phage and bacterial titers were stable. (**B**) SU57 produced a forceful infection with drastic changes in both PFUs and CFUs; however, this effect faded away over time. (**C**) ECO57 sequentially treated with SU10 first then SU57. (**D**) ECOR57 sequentially treated with SU57 first and then SU10.

In order to determine when phage resistance arises, it is necessary to understand the population structure of bacteria that have been infected by single and multiple phages. Infecting bacterium ECOR57 with either of the SU10 or SU57 phages at different MOIs produced phage-specific characteristic population dynamic curves and caused specific growth responses within the bacterial population. The initial concentration of phage and bacteria at infection in these experiments was between 10^9^ and 10^10^ plaque forming units (PFUs) or colony forming units (CFUs)/mL. A steady state was reached approximately after 48 h, with phage titers of approximately 10^10^ for SU10 and 10^7^ PFU/mL for SU57, and bacterial titers of approximately 10^9^ CFU/mL. Phages and bacteria coexisted for the entirety of the experiment, 120 h after infection. SU10 alone resulted in a moderate production of phages in ECOR57, where phage and bacterial titers eventually stabilized (Figure 1A). Additionally, infection by SU10 resulted in a relatively long latent period of 45 min. The SU10 phage titers oscillated between 10^8^ and 10^11^ PFU/mL with the lowest titers observed 2–3 h after infection and the highest titers observed 6–7 h after infection. Phage SU57, on the contrary, produced a faster infection in ECOR57, with drastic changes in phage and bacterial numbers (dropping from 8 × 10^8^ to 4.3 × 10^6^ CFU/mL after the first 30 min of infection) (Figure 1B). The SU57 phage titers oscillated between 10^6^ and 10^11^ PFU/mL, with the maximum titer three hours after infection and minimum titer 24–32 h post-infection. Following SU57 infection, the bacterium ECOR57 exhibited titer changes between 10^5^ and 10^10^ CFU/mL, with the minimum titer seen approximately 3–4 h post-infection and maximum titer seen after 24 h. Infection by SU57 caused an initial drastic drop in the ECOR57 titer while, at the same time, exponentially increasing its own phage titer. This effect was very strong at the beginning of infection and faded away over the experimental duration (Figure 1A,B and Figure S1). In general, the combination of SU10 and SU57 infecting bacterium ECOR57 gave similar population dynamic outcomes as observed for each individual phage, irrespective of whether SU10 or SU57 was given first, suggesting that each phage acts independently of each other. However, the SU10 titers were indirectly affected by the low bacterial titers resulting from SU57 primary infection (Figure 1C,D and Figure S1).

**Figure 2:**
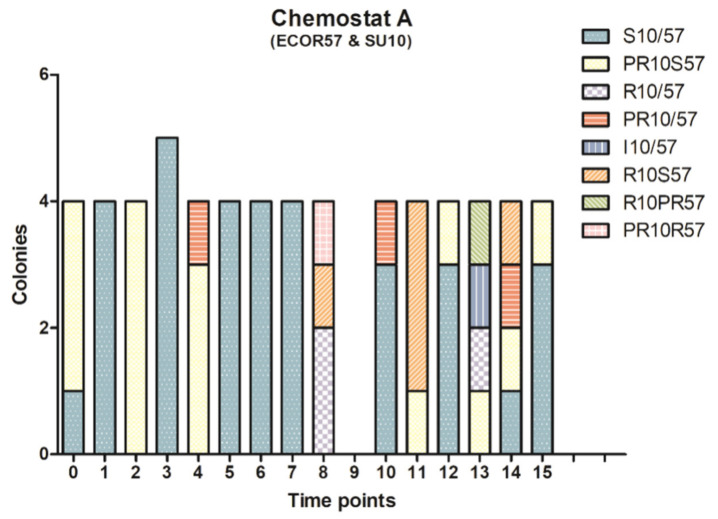

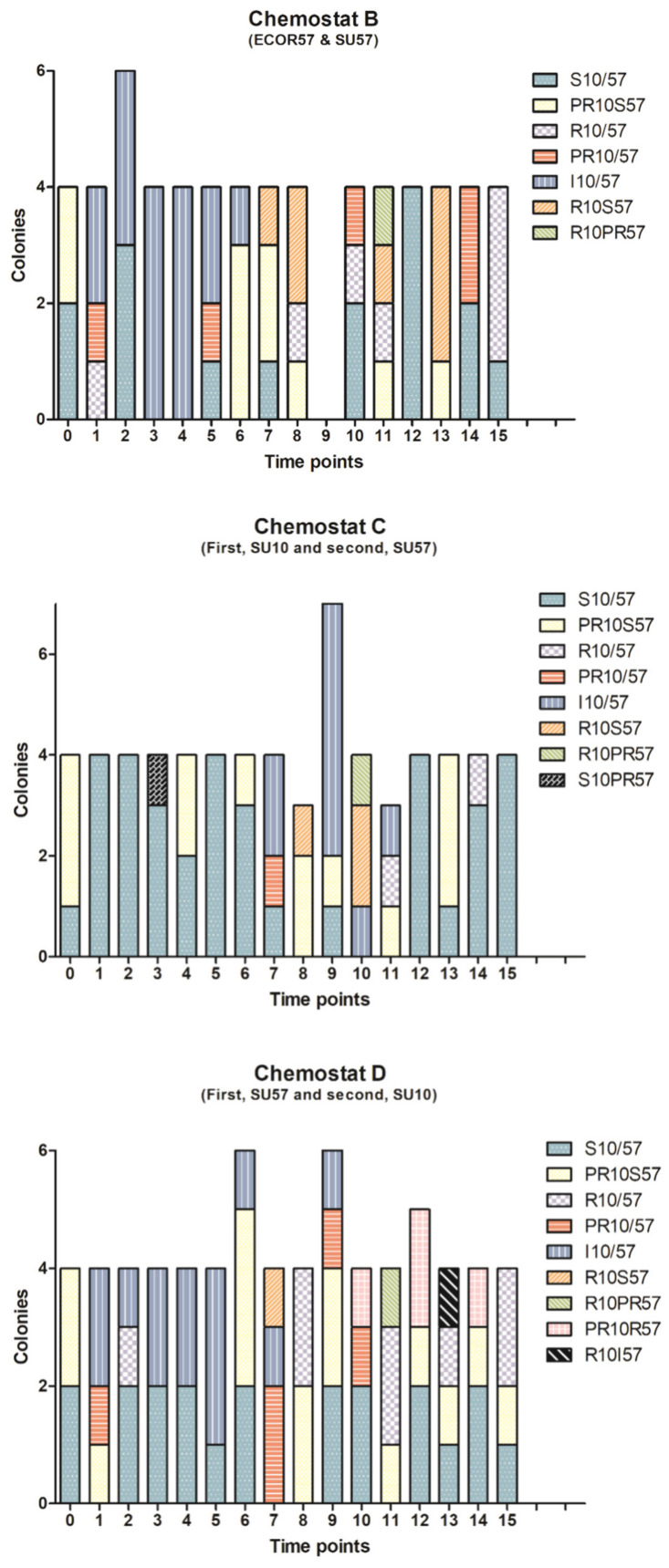
Resistance/susceptibility patterns of the bacterial population following one or two-phage infections. Using EOP, during early infection, all bacteria were susceptible (S) to phage infection with resistance (R) arising in most bacteria towards later time points. A mix of S, R, partial resistant (PR), and those bacteria whereby resistance could not be determined (inconclusive; I) were seen during intermediate time points. The bacterial population had more pronounced changes when SU57 was present compared to SU10. Over a span of 16 sampling points (T = 0, 0.5, 1, 1.5, 2, 3, 4, 6, 24, 25, 26, 28, 30, 47, 53, and 72.5 h), a total of 260 colonies were tested (61 from chemostat A (**A**), 62 from chemostat B (**B**), 66 from chemostat C (**C**), and 71 from chemostat D (**D**)).

Following phage exposure, ECOR57 bacteria in each chemostat were collected at 16 different time points (T = 0, 0.5, 1, 1.5, 2, 3, 4, 6, 24, 25, 26, 28, 30, 47, 53, and 72.5 h) and analyzed for resistance/susceptibility against phages SU10 and SU57 using efficiency of plating (EOP). The number of colonies analyzed at each time point were randomly selected. A trend whereby ECOR57 was sensitive to phage adsorption during early experimentation and developed resistance during later experiment time points was observed (Figure 2). A mix of phage sensitive (identified as having clear phage plaques covering the whole plate without having complete lysis), resistant (a lawn of bacteria with no observed phage plaques), partial resistant (a lawn of bacteria having between one and approximately 1000 phage plaques), and those which were inconclusive was present during intermediate experimental time points. Bacteria exposed to SU57 exhibited a faster resistance development compared to SU10-exposed bacteria, irrespective of SU10’s long latent period. In an attempt to quantify the mutational rates of resistance against SU10 and SU57, modified Luria–Delbrück fluctuation tests were performed [41,46], but the results were inclusive. The mutational rates could not be calculated, as there were no sensitive bacteria observed, i.e., growth in all wells, which could be attributed to the phenotypic and partial resistances observed. Cross-resistance was also observed whereby the presence of one phage caused ECOR57 bacterium to be resistant to both phages. Such bacteria were found irrespective of whether SU10 or SU57 was the primary infecting phage; bacteria resistant to SU57 appeared in chemostat A where ECOR57 was only treated with SU10, and SU10-resistant bacteria were observed in chemostat B where ECOR57 was only treated with SU57 (Figure 2). It is worth noting that no colonies completely resistant to SU57 and susceptible to SU10 were found. 

### 2.2. Phenotypic Diversity among ECOR57 Bacteria following Exposure to Two Phages

**Figure 3:**
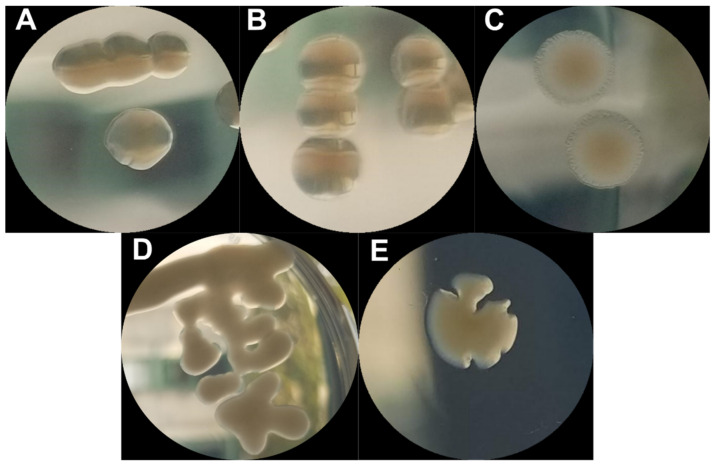
Phenotypic diversity among ECOR57 bacteria following infection by SU10 and/or SU57: (**A**) wild-type ECOR57; (**B**) the normal morphology with smooth edges and semi-glossy finishing; (**C**) the edgy morphology exhibited large flat colonies with a halo resembling a fried egg; (**D**) the slimy morphology had a mucoid surface with very smooth edges and a high gloss–shiny finish; (**E**) the pointy morphology had irregular colony edges with sharp divots.

Upon sampling ECOR57 bacteria from each chemostat following phage exposure, four distinct colony morphotypes (termed normal, slimy, edgy, and pointy) were observed (Figure 3 and Figure S2). Those that exhibited a normal phenotype resembled that of wild-type ECOR57 with round smooth edges and semi-glossy surfaces. Bacteria with white mucoid surfaces, very smooth edges, high gloss, and a shiny finish were termed slimy. Large, flat, dry, and rough looking colonies resembling a fried egg were termed edgy. Colonies that exhibited irregular edges with sharp divots were termed pointy and could not form a uniform bacterial lawn. These bacteria had either total lysis or a granular appearance, even in the absence of phage. ECOR57 bacteria exhibiting these four morphotypes did not revert back to its wild-type morphotype upon re-streaking. Preliminary genomic analyses confirmed that the observed morphotypes were indeed variants of ECOR57 and not the result of contamination (Supplementary Materials Figure S3). 

**Figure 4:**
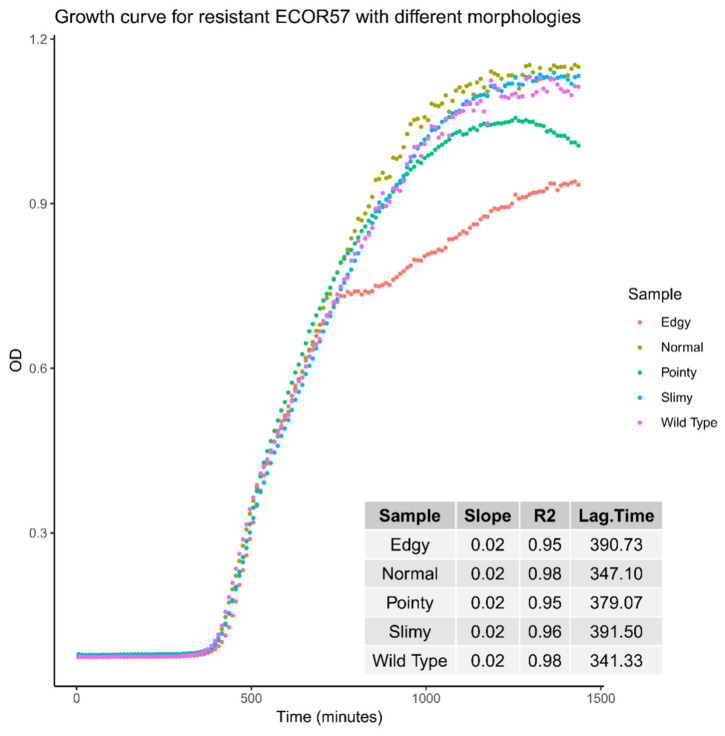
Growth curves for ECOR57 exhibiting different morphologies following phage infection. The combined growth curves of the different morphologies were obtained from the BioScreen Analyzer. All bacteria seemed to follow the same trend with the normal and slimy morphologies following closely to the wild type. Those exhibiting the edgy morphology seemed to reach a stationary phase faster than the other morphologies, with a lag time similar to those with the slimy morphology, an approximately 50 min difference from that of the wild type. Samples were run as technical quadruplets and experiments were performed in triplicate.

When comparing the growth curves of wild-type ECOR57 and the various morphotypes over 24 h, the following was observed: Those with the normal phenotype had the shortest lag time similar to the wild type (approximately 347 vs. 341 min), those with the edgy and slimy morphologies had a similar lag time with the longest duration (approximately 391 vs. 392 min), and those with the pointy morphology had a lag time of approximately 379 min. Bacteria presenting the edgy morphotype reached a stationary phase earlier, approximately 10 h after starting the growth experiment, compared to the other morphotypes and wild-type ECOR57 bacteria (Figure 4).

**Figure 5:**
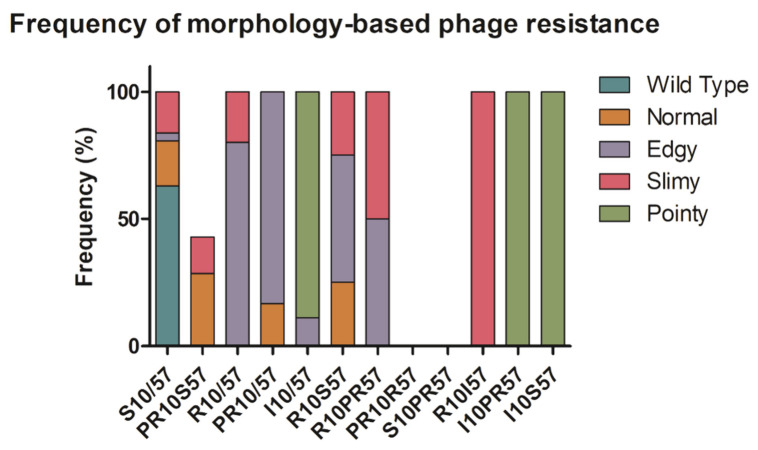
Frequency of morphology-based phage resistance. Using EOP assays, resistance (R) was determined as a lawn of bacteria with no observed phage plaques; susceptibility (S) as having clear phage plaques covering the whole plate without having complete lysis; partial resistance (PR) as having between 1 to approximately 1000 phage plaques present; or determined inconclusive (I). A total of 99 colonies were tested of which 40 colonies represented the wild type; 16 colonies represented the normal, slimy, and edgy morphologies; 11 colonies represented the pointy morphology.

The EOP of phages were performed in order to determine whether these various morphotypes provided resistance against phage infection. Bacteria with the normal and edgy morphotypes showed the highest frequency or resistance (including partial resistance) to infection by phages SU10 and/or SU57. Those with the slimy phenotype had the second highest resistance frequency. For those with the pointy morphotype, resistance and susceptibility frequencies were inconclusive (Figure 5). 

**Figure 6:**
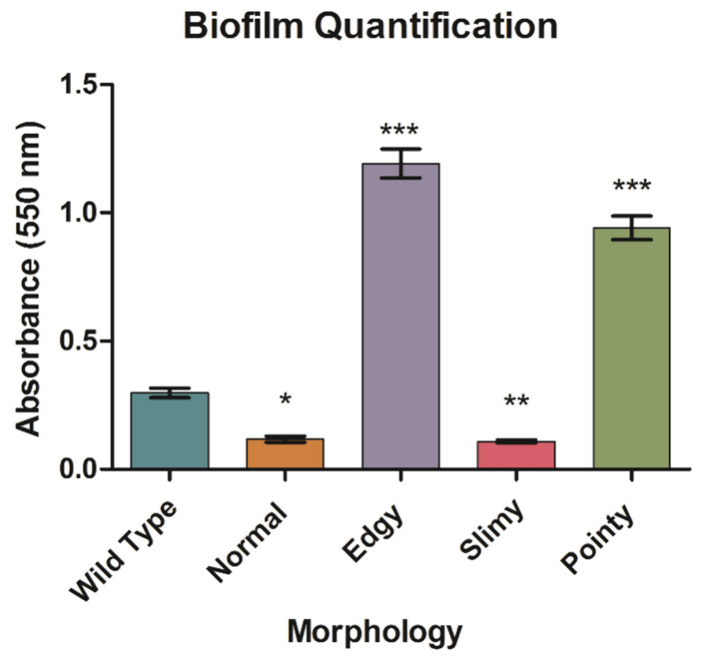
Quantification of biofilm arising from the different morphologies. Biofilm from bacteria representing the different morphologies (i.e., normal, slimy, edgy, and pointy) were evaluated using crystal violet staining after overnight growth and optical density readings at 550 nm of the solubilized crystal violet. The results show the mean of three independent experiments run in quadruplets. Error bars represent the SEM of each run. One-way ANOVA with post hoc Tukey, *n* = 5, was performed and significance compared to the wild type was set at *p* < 0.05 (*), *p* < 0.01 (**), and *p* < 0.001 (***).

Biofilm formation from the various ECOR57 morphotypes were quantified, since biofilm production is a way to confer resistance against phage adsorption by hiding phage receptors on its surface. Compared to wild-type ECOR57 bacteria, there was an overall significant difference in the amount of biofilm formed by these four morphotypes. Of the four, the edgy and pointy morphologies produced statistically significant (*p* ≤ 0.001 using one-way ANOVA with post hoc Tukey; *n* = 5) amounts of biofilm, with the edgy generating higher amounts than the pointy morphology (Figure 6). It is worth noting that when comparing the various morphologies to each other, there were statistically significant (*p* ≤ 0.001) differences in the amount of biofilm formed, except between the normal and slimy morphologies. Using antibiotic disk diffusion assays, the correlation between the varying ECOR57 bacterial morphotypes, including those presenting double resistance, and their susceptibility to cell-wall targeting antibiotics, such as ampicillin, imipenem, and ceftazidime, proved negative (Supplementary Materials Table S1).

## 3. Discussion

The human gastrointestinal tract is a complex environment that has an oxygen concentration, pH, and nutrient availability that influences the quantity and types of bacteria present [3]. Phages play a role in gut homeostasis, influencing bacterial diversification by providing constant infection pressure [3,4]. The use of added phages to modify the type and quantity of bacteria present in the gut would be ideal from a therapeutic standpoint. However, bacteria can develop resistance mutations against single-phage infections by modifying cell surface receptors, which often affects cell membrane integrity, nutrient transport, or mobility [28,47,48]. Despite this, little is known about the influence that multiple phage infections have on a single host’s anti-phage defense system. Resistance against combinations of phages is dependent on the overall population dynamics between phages and host, the types of and concentrations of phages used for infection, and the order that phages are added [28]. This would possibly require more general phage-resistant systems than, for example, systems targeting specific sequences of incoming DNA. 

### 3.1. Population Dynamics and Resistance Patterns of a Two Phages–One Host System

Chemostat experiments revealed that both SU10 and SU57 produced phage-specific growth curves. Infection by SU10 resulted in a moderate production of phages, where both phage and ECOR57 bacterial titers were stable. One possible explanation for this lies with SU10’s infection efficacy. When infecting ECOR57, SU10 had an adsorption rate of 1.43 × 10^−9^ mL/min, almost half the rate of when it adsorbed to its host bacteria of ECOR10 (3.10 × 10^−9^ mL/min) (Supplementary Materials Figure S4). SU57 on the other hand, caused a more dynamic infection with large oscillations in phage and bacterial titers. A possible explanation for these initial killing curves lies in the individual phage kinetics used for infection. SU10 is a C3 phage belonging to the *Podoviridae* family [45,49], while SU57 is a T1-like phage belonging to the *Drexlerviridae* (formerly *Siphoviridae*) family [35]. Compared to SU10’s burst size of 166 PFUs and latent period of 45 min [49], SU57 had a smaller burst size of 13 PFUs and a shorter latent period of 14 min. It also had an adsorption rate constant of 8.5 × 10^–10^ mL × min^–1^ [35]. Like its T1 and T1-like phage relatives [50,51], SU57 produced large clear plaque. This could be indicative of efficient bacteriolytic proteins, such as endolysin, being made by SU57 compared to SU10 [35,49]. With these properties, it is possible that the phage SU57 is more virulent when infecting ECOR57, mounting a more productive infection than SU10. It is also worth noting that despite SU10’s ability to infect ECOR57, its infection was not to the same degree as it was on its host of isolation ECOR10 bacteria. Exposure to various MOIs of phage could also explain the initial killing rates in the population dynamic curves. From a population perspective, high MOI exposure could cause faster phage growth and bacterial lysis since more cells will be infected by at least one phage. However, within the population, there will also be cases of bacterial cells being infected by more than one phage. If a successful infection occurs (i.e., all phages infecting the same cell successfully start to replicate), it is speculated that longer latent periods and larger burst sizes will be observed due to the increased load on the bacterial transcription and translation machinery [35,52]. It is important to note that a coinfection or co-adsorption by two phages of the same phage type would result in a different outcome compared to a coinfection by two different phage types where the latter could result in smaller burst sizes and possibly an infection exclusion of one of the phages [53]. This would, in turn, affect the rate of phage progeny production, the rate of bacterial lysis, and rate of bacterial–phage resistance development (i.e., the rate at which bacteria develop resistance mechanisms to prevent phage infection and, in turn, the rate at which phages develop countermeasures to these bacterial-resistance mechanisms). 

Previous studies have shown that the effect of a combinational phage infection depends on the order and timing of the addition of phages to a bacterial system [28]. Phage-specific curves were still seen when ECOR57 bacteria was treated sequentially with SU10 and SU57. Sequential treatment of SU57 followed by SU10 also produced phage-specific curves; however, there was a small difference in the SU10 phage titers. The decrease in SU10 titer likely resulted from the byproduct of a faster infection with SU57. This faster infection could have killed off a portion of the bacterial population resulting in a smaller number of bacteria available to be infected by SU10 (as observed by the bacterial counts). Another possible explanation for SU10’s lower titer could be its adsorption rate and latent period compared to SU57. Although SU10 is approximately 50% faster in adsorption, its latent period is three times as long as SU57. With SU57 being able to produce progeny faster than SU10, this could influence the competition for receptors on ECOR57’s cell surface, thereby affecting SU10 adsorption and contributing to its lower phage titer. 

One of the properties that makes phages an interesting candidate to treat antibiotic-resistant infections is auto-dosing [54,55]. Under ideal conditions, phages should be able to decrease the bacterial count in a dose-dependent manner and be able to decrease the bacterial count until there are no longer any viable hosts to infect and produce progeny, whereby a decrease in phage titer is expected. However, the two phages–one host population dynamic curves depicted the opposite. Towards the end of the experiments, ECOR57 bacteria and phages seemed to reach an equilibrium where both bacteria and phages survived. It is possible that bacterial survival can be attributed to the development of resistance mechanisms among bacteria [31]. Over time and under pressure from single-phage infections, bacteria are capable of going through a resistance spectrum, moving from susceptibility, gradually to partially resistant and, eventually, to completely resistant. This can be conducted by producing surface modifications to prevent phage binding and adsorption [28,56,57]. The fast infection kinetics by SU57 impose a strong selection on ECOR57 bacteria to develop phage resistance faster compared against SU10, which can be observed as the appearance of early resistance development (Figure 2). These resistance mutations can then pass through the bacterial population at a high frequency [29]. The presence of such resistance mutations could be an explanation as to why the mutational rates of ECOR57 against SU10 and SU57 could not be ascertained. Interestingly, the phenomenon of cross-resistance was observed in chemostats A and B, where ECOR 57 was treated with either SU10 or SU57. This cross-resistance phenomenon was most likely a contributing factor to the population growth curves’ appearance when the phages were added sequentially (chemostats C and D). Recently, antibiotic tolerance has been described as a novel strategy by which bacteria can overcome antibiotic treatment by temporarily enduring or slowing the bactericidal effects of antibiotics without being able to grow in their presence [58]. Perhaps the same tolerance phenomenon is exhibited by bacteria exposed to phages, which could account for the growth curves and partial resistances observed in ECOR57. Additionally, phage tolerance could explain the difficulty in determining the ECOR57 mutational rate under SU10/SU57 phage infection. Like antibiotic tolerance, phage tolerance could accelerate the evolution of genetic resistance by increasing the likelihood for resistance mutations arising within a bacterial population [58].

“Leaky resistance”, on the other hand, could be an explanation for the phage survival observed [59]. Phage-resistant bacterial cells usually become the dominant population of bacteria, whose growth is limited by the amount of resources present and degree of cell membrane integrity stability [59]. However, there is a small proportion of the population that remains in a stable state that is susceptible to phage infection [59]. These susceptible bacteria then maintain the phage population by continuously producing new phage progeny. Another explanation for this phage survival could be a state of latent viral infection, or more simply put, a sustained phage infection where the phage is not cleared by the bacteria but remains within infected bacterial cells. It is possible that during a sustained infection, there could be both a silent and productive infection that occurs without rapidly killing or inducing excessive damage to the host bacterial cell [60]. The probability of a sustained phage infection occurring would be dependent on how well the phage initiates infection (i.e., the adsorption rate). 

### 3.2. Phenotypic Diversity within a Two Phages–One Host System

In addition to the development of single resistances and cross-resistance mechanisms, the ECOR57 bacteria underwent phenotypic changes under pressure from phage infection. Infected ECOR57 bacteria underwent phenotypic changes that bred true following multiple re-streaks (Figure 3). Previous studies have shown that phage-resistant bacteria often display colonies that are rough and dry, lacking many surface proteins [61,62] or mucoid [63]. To our knowledge, the pointy morphology has not been observed frequently. Different resistance frequencies (i.e., susceptible, partially resistant, resistant, or inconclusive) were observed for each morphotype seen. Though beneficial against phage infections, these mutations could result in bacteria having fitness costs, such as decreased nutrient uptake and slower growth rates [64], as was evident by those displaying the edgy and pointy morphologies. 

The various colony morphotypes observed could be the result of phase variation [65]. Phase variation refers to the reversible switching between an “on/off” expression state, whereby the level of protein expression varies among individual cells of a population [66]. Hasman et al. (2000) observed the influence of two-phase variable surface structures: antigen 43 and type 1 fimbriae in *E. coli* strain K12 [67]. Large, flat, frizzy colonies were seen upon upregulation of antigen 43 and a small glossy colony morphology upon the upregulation of type 1 fimbriae [67]. It is possible that under SU10 and/or SU57 phage duress, the ECOR57 bacteria could upregulate these surface structures, resulting in the edgy and normal morphologies observed. Phase variation has also been observed to affect the strength of bacteria–bacteria interactions [68,69]. Modulation of type IV pili in *Neisseria gonorrhoeae* has been shown to affect the shape of early biofilm formation and as well as the standing variation of bacterial populations that affect bacterial positioning and competition dynamics within a bacterial colony [69,70]. This could explain the biofilm formation observed by those bacteria with the edgy and pointy morphologies. This could also explain the pointy colony morphology, whereby the divots formed are slow growing, weakly interacting cells along the expanding front of fast growing, strongly interacting cells of the same colony. However, mutational and knockout studies of these surface structures would be needed to make such conclusions.

The mucoid shiny appearance of bacteria can be attributed to the overproduction of exopolysaccharide, the main EPS component found in many biofilms [71,72]. This mucoid confers phage resistance by acting as a physical barrier between the cell surface receptor and the infecting phage [48,63,73]. It was expected that those exhibiting the slimy morphology would produce the most biofilm and have a high rate of resistance development. However, this was not the case; it was the edgy morphology that formed a higher amount of biofilm and exhibited a higher frequency for developing resistance. Those presenting the edgy morphotype could have an increased expression of curli fimbriae and cellulose matrix components, as is typical of Enterobacteria presenting the red, dry, and rough (Rdar) phenotype [74,75]. The Rdar phenotype is dependent on the master transcriptional regulator CsgD [74,76,77], which controls the expression of *csgBA* (encoding the structural subunits of curli fimbriae) [76,78] and *adrA* (encoding for cellulose biosynthesis) [76,79]. CsgD is also positively modulated by bis-(3′-5′)-cyclic dimeric guanosine monophosphate (c-di-GMP) [80], whereby an increased amount of c-di-GMP is associated with sessility and biofilm formation [74,76]. It is possible that bacteria presenting the edgy morphotype exhibited CsgD and c-di-GMP upregulation; however, these were not tested for. Mucoidy, on the other hand, could explain the phage–bacteria equilibrium observed. Under the protection of mucoidy, some mucoid phage-resistant mutants have a high rate of reversion to a non-mucoid phage-sensitive type when phages are removed or can no longer infect [63]. This allows for a small proportion of bacteria to be capable of influencing both phage numbers (i.e., leaky resistance) and bacterial numbers.

## 4. Materials and Methods

All materials utilized and experiments performed were treated and carried out using standard microbiological laboratory practices in order to reduce any contamination risks.

### 4.1. Bacterial Strains and Growth Conditions

The bacterial strains used for this paper were ECOR10, ECOR57, and ECOR63 from the ECOR standard reference collection of *E. coli* [44], which was generously supplied by Diarmaid Hughes and Dan Andersson of Uppsala University, Sweden. The bacteria were either cultured in liquid lysogeny broth (Miller LB; Neogen, Lansing, MI, USA) or on tryptone yeast agar (TYA; Biolife Italiana, Milano, Italy) plates. Overnight bacterial cultures were grown at 37 °C with shaking at 150 RPM and plates were statically incubated at 37 °C. For comparative bacterial analyses and phage tittering, fresh LB media was inoculated with overnight bacterial cultures and allowed to develop to mid-log phase at 37 °C until optical density at 600 nm (OD_600_) reached 0.6. 

### 4.2. Phage Propagation, Purification, and Quantification

Phages SU10 (*Podoviridae* with C3 morphotype) and SU57 (T1-like *Drexlerviridae* with B1 morphotype), previously isolated from Käppala water treatment plant 15 km East of Stockholm, Sweden [49], were obtained from stocks based at Stockholm University. High titer phage stocks of SU10 and SU57 were made using a modified polyethylene glycol (PEG) precipitation procedure [81]. Briefly, crude phage lysate was centrifuged at 3864× *g* for 10 min and the supernatant was filtered through 0.45 µm sterile syringe filter (Sarstedt Filtropur, Nümbrecht, Germany). Phages were precipitated by adding solid NaCl and PEG8000 (Acros Organics, Schwerte, Germany) to the partially purified suspension to have a final concentration of 1 M NaCl and 10% (*w*/*v*) PEG8000 and stored at 4 °C for two weeks. Phages were recovered through centrifugation at 11,000× *g* for one hour at 4 °C. The phage pellet was re-suspended in 50 mL phosphate buffered saline (PBS) and stored at 4 °C until experimentation. Phage quantifications, measured as PFU/mL, were determined using the overlay agar (OA) method consisting of 65% (*w*/*v*) (22.775 g/L) TYA [82,83]. In short, three mL of OA was inoculated with 100 µL of overnight host bacteria culture (approximately 10^8^ CFU/mL) and 100 µL of serially diluted phage in PBS. The OA was poured over pre-prepared TYA plates and incubated at 37 °C for at least 18 h. The stock titer for SU10 was 4.1 × 10^10^ PFU/mL and 7.5 × 10^11^ PFU/mL for SU57.

### 4.3. Chemostat Setup

Homemade chemostats (Supplementary Materials Figure S5) were constructed and assembled according to Cornejo et al., (2009) [84]. Silicone-peroxide tubes (1.6 or 3.2 mm) connected a LB reservoir (5 or 8 L) to four different chemostats (20 mL total reaction volume), which were also connected to a waste flask. The reservoir and each of the chemostats were set up to have aeration tubes fixed with 0.2 µm filters (Sarstedt Filtropur, Nümbrecht, Germany) to allow for air flow and to avoid contamination. The entire chemostat set up (including the LB reservoir and waste flask) was sterilized via autoclaving before experimentation. A peristaltic pump (IPC high precision multichannel dispenser) was used to drive the LB from the reservoir to the chemostats and a vacuum pump was used to discard waste into the waste flask. The flow rate used was 0.196 mL min^−1^. 

ECOR57 bacteria were inoculated into the chemostats and was left to grow for 24 h in a 37 °C water bath until they reached steady state. Phages SU10 and/or SU57 were added at different time points and different MOIs in order to investigate changes in population dynamics between phages and bacteria. Five hundred microliter aliquots of the phage(s)–bacteria mixture were removed at 16 specific time points (T = 0, 0.5, 1, 1.5, 2, 3, 4, 6, 24, 25, 26, 28, 30, 47, 53, and 72.5 h) for quantification and characterization (as described below). Throughout experimentation, the following conditions were kept: Chemostat A contained ECOR57 and SU10, Chemostat B contained ECOR57 and SU57, Chemostat C contained ECOR57 which was first infected with SU10 and then with SU57, and Chemostat D contained ECOR57 which was first infected by SU57 and then by SU10. The MOIs tested for SU10 were 0.33, 0.42, 0.43, 0.50, 0.55, 0.65, 0.83, 1.00, 1.10, 1.50, 2.30, and 1300. The MOIs tested for SU57 were 0.13, 0.12, 0.16, 1.04, 1.14, 1.20, 3.40, 3.60, 4.50, 10.4, 25.9, and 46.9. ECOR57 bacteria were quantified using the drop count methodology [85]. SU10 and SU57 phage titers were determined using the previously described methodology. The following indicator bacterial strains were used to determine the phage titers: in chemostats A, C, and D: ECOR10 to measure phage SU10 as it is permissive to SU10 and refractory to SU57; chemostat B: ECOR57 to measure phage SU57. ECOR63 bacteria was used to titer phage SU57 in chemostats where multiple infections were occurring (i.e., chemostats C and D) as it is permissive to SU57 and refractory to SU10. The experimental duration for each chemostat run was 72.5 h. Chemostat experiments were performed four times (see Supplementary Materials Figure S1 for the additional three experiments).

### 4.4. Determination of Adsorption Rate Constant

The adsorption rate constant of SU10 on bacteria ECOR10 and ECOR57 were determined using the modified one-step growth curve protocol used by Koonjan et al., (2020) [35]. In summation, 50 mL of LB was inoculated with either 50 µL of ECOR10 or ECOR57 and incubated at 37 °C with shaking until the bacteria reached the mid-log phase (OD_600_ 0.6). Once OD_600_ 0.6 was reached, bacterial suspension was removed; the final volume of ECOR10 bacterial suspension used for experimentation was 44.991 mL and the final volume of ECOR57 was 44.9933 mL. A total of 9 µL of SU10 phage stock (2.85 × 10^10^ PFU/mL) was added to mid-log phase ECOR10 bacteria (approximately 3.95 × 10^7^ CFU/mL) at a multiplicity of infection (MOI) of 0.144 and mixed by swirling (T = 0). One microliter of aliquots, withdrawn every five minutes for an experimental duration of 25 min, were centrifuged at 6000× *g* for one minute. Using the supernatant, 1:10 serial dilutions in PBS were done to determine the phage titer at each time point. All experiments were done in triplicates. This procedure was repeated for ECOR57 bacteria whereby, 6.7 µL of SU10 phage stock was added to mid-log phase ECOR57 bacteria (approximately 3.70 × 10^7^ CFU/mL) at a MOI of 0.107. The adsorption rate constants for both ECOR10/SU10 and ECOR57/SU10 infection were determined using the following formula, where *N* is the bacterial density, *Po* and *P* are the starting and ending phage titers, *k* is the adsorption constant, and *t* is the time in minutes over which adsorption occurs:

$$k=-\ln\left(P/Po\right)/Nt$$

It should be noted that the adsorption rate constants were determined using only two time points (T = 0 and T = 5).

### 4.5. Efficiency of Plating (EOP) and Frequency of Phage Resistance Development

The frequency of phage resistance incidence was determined using EOP plaque assay method. Previous experiments showed optimal phage plaques when the phage stocks were diluted to 10^−4^ for SU10 and 10^−6^ for SU57. As such, these dilutions were used to test the frequency of bacterial resistance development. One hundred microliters of overnight bacterial culture was used to inoculate OA containing 100 µL of either 10^−4^ SU10 dilution or 10^−6^ SU57 dilutions. LA plates containing the OA were incubated overnight at 37 °C. Phage resistance was based on the number of plaques observed rather than based on plaque morphologies. Resistance was determined as a lawn of bacteria with no phage plaques observed. Susceptibility was determined by having clear phage plaques covering the plate without having complete bacterial lawn lysis. Partial resistance was assessed as having between 1 to approximately 1000 phage plaques present. 

### 4.6. DNA Extraction, Sequencing, Bioinformatics, and Genomic Analyses

In order to confirm that the various morphotypes observed were indeed ECOR57 and not contamination, preliminary genomic analyses were carried out. The genomic DNA of wild type ECOR57 and all its variants were extracted using DNeasy Blood & Tissue Kit (Qiagen, Hilden, Germany) according to the manufacturer’s protocol specific for bacterial DNA extraction. The genomes were sequenced using Illumina Miseq™ sequencing system at Stockholm University, The Wenner–Gren Institute, Stockholm, Sweden. The raw reads were trimmed using Trimmomatic version 0.36 and assembled using SPAdes [86,87]. Open reading frames and genes were predicted and annotated using PROKKA [88]. Preliminary phylogenetic analyses showing the relationship between wild type ECOR57, its variants, the most commonly used laboratory strain of *E. coli* (strain K12-MG1655; NCBI accession number NC_000913.3) and three other *E. coli* strains (NCBI accession numbers EGK5463891.1, EET2940696.1, and STN07265.1) were performed using the *E. coli* housekeeping gene *rpos*, which encodes for the RNA polymerase sigma factor RpoS [89,90]. Alignments were made in ClustalW with default settings embeded in the Mega-X software and in MAUVE version 20150226 [91,92,93]. A neighbor-joining phylogenetic tree was constructed using Mega-X with default settings. 

### 4.7. Bacterial Growth Analyses

The growth curves for ECOR57 wild type bacteria and the different morphologies observed for phage resistance were determined using BioscreenC (Bauer Core, MA, USA). LB was inoculated with 100 uL of overnight bacterial culture and allowed to reach mid-log phage (OD_600_ 0.6) with 37 °C shaking incubation. Optimal bacteria concentration used for the BioscreenC analyses was assessed by the drop count method [94]. In brief, bacteria cultures were serially diluted 1:10 in PBS. 3 × 10 µL drops from dilutions 10^−4^ to 10^−7^ were plated in duplicates and incubated at 37 °C. The dilution which gave between 3–30 individual colonies was used. Using this dilution for all bacterial samples (for this experiment it was 10^−6^), 100 µL was used to inoculate 900 µL fresh LB, of which 150 µL was loaded onto the 100 well BioscreenC honeycomb plate. All samples were run as technical quadruplets and experiments were performed in triplicates. Each run included PBS negative controls and LB blanks. The BioscreenC EZ Experiment Software had the following settings: 37 °C incubation temperature, 600 nm filter, three-day experiment duration, and 10-min measurement interval with continuous shaking and medium amplitude. Growth curves and growth rate calculations were carried out using R [95,96]. 

### 4.8. Luria–Delbrück Fluctuation Tests

In order to quantify the mutational rates of ECOR57 resistance against SU10 and SU57, Luria-Delbrück fluctuation tests were performed. Modified from Borin et al., (2021) Lang (2018), and Luria and Delbrück (1943), the following was performed: An overnight culture of ECOR57 (initiated from a single colony) was diluted such that each well of a 96 well plate contained 200 µL of approximately 10^3^ CFU/mL. The plate was incubated overnight at 37 °C with shaking. Following incubation, the cultures were diluted to have 10^6^ CFU/mL using PBS. A 50-well master mix for each phage was made (containing 2.5 mL of either SU10 or SU57 phage and 7.5 mL of LB) from which 200 µL was dispensed per well (40 wells per phage). 10 µL of bacteria diluted to have 10^6^ CFU/mL were added to each well. Using the SpectraMax i3x system, the plate was incubated at 37 °C with shaking for 20 h and the bacterial growth was measured using OD_600_. The mutation rate (µ*_r_*) was calculated using the *P*_0_ method:

$$\left(P_{0}=e{\;}^{{µ}_{r}*\left(N-N_{0}\right)}\right)$$

where *P*_0_ is the proportion of wells that were sensitive (wells that exhibited no growth), *N* is the number of cells inoculated in each well, and *N*_0_ is the number of cells used to start cultures (approximately 10^3^ CFU). Fluctuation experiments were replicated in triplicate. 

### 4.9. Antibiotic Resistance Testing

Phage-resistant ECOR57 bacteria exhibiting different morphologies and phage-resistance patterns were re-suspended in PBS until OD_600_ was approximately 0.06 (equivalent to McFarland standard 0.5) and plated on Mueller Hinton broth II agar plates (Sigma–Aldrich, Taufkirchen, Germany). Antibiotic resistance/susceptibility were determined using the European committee on antimicrobial susceptibility testing (EUCAST) antimicrobial disk diffusion zone diameter breakpoints for the following antibiotics: 10 µg imipenem, 10 µg ampicillin, and 10 µg ceftazidime (Thermo Scientific Oxoid, Wesel, Germany). Plates were incubated at 37 °C overnight, after which zones of inhibition were measured. Samples showing ampicillin resistance were further tested on Mueller Hinton broth II plates containing different concentrations of ampicillin (1:1, 1:2, 1:4, 1:8, and 1:16). 

### 4.10. Biofilm Formation and Quantification

Biofilm production for the various morphologies of ECOR57 was determined using a modified protocol from O’Toole (2011) [97]. Overnight bacterial culture was diluted 1:100 in fresh LB of which one mL was added to 24-well plates (Corning Multiwell 24 well) in duplicates. Plates were incubated at 37 °C for 24 h. After 24 h, the media was removed and washed twice with PBS to remove excess bacterial debris and media components. 250 µL of 0.1% Crystal Violet (BD Gram Crystal Violet) was added to each well and allowed to stain for 15 min at room temperature. After crystal violet removal, wells were washed approximately four times or until the washing fluid was no longer purple. Plates were allowed to dry in a 37 °C incubator for approximately five minutes. To quantify the biofilm, 250 µL of 30% acetic acid in water was added to each well to solubilize the biofilm and allowed to sit for 10 min. Fifty microliters of each sample was transferred to round bottom 96 well microtiter plates (Corning 96-well cell culture cluster round bottom) in quadruplets. Optical density at 550 nm was measured using BMG LabTech POLARstar Omega plate reader. One-way ANOVA with post hoc Tukey statistical analysis with *n* = 5 was performed using GraphPad Prism version 5.0.

## 5. Conclusions

Within the human gut microbiome, interactions between phages and bacteria are inevitable. In a two phages–one host system, the types of phages, their individual phage kinetics, and MOI used for infections influenced their infection efficacy and growth on a single bacterial host strain. Additionally, over time and under pressure from phage infection, bacteria were prone to developing a range of resistances (varying from susceptible to partial resistant to completely resistant) and underwent phenotypic changes most likely due to the phage tolerance, “leaky” resistance, and phase variation. 

While these experiments provide insight into the mechanics of a two phages–one host system, the pervasiveness of these interactions within the human gut microbiome remains largely unknown. The two phages–one bacteria interactions in the gut could ultimately dictate whether it will be possible to use solely phage cocktails therapeutically against the same bacterium or if, instead, complementary to antibiotics [48,98,99]. Future experiments encompassing sequencing and genomic analyses could provide an additional component to unveiling the processes responsible for the observed population dynamics in a two phages–one bacterial host system. 

This study provides the foundation and a way to observe and quantify different phage(s)–bacteria combinations. Phages intended for therapeutic usage must be further researched and characterized in such a manner in order to elucidate their infection kinetics in combination with other phages, the coevolution of resistance mechanisms by both phages and exposed bacteria, as well as the dosing to reach the site of infection and produce an active infection. 

## Data Availability

The sequencing data used to show that there was no contamination and the various morphoptypes observed were indeed variants of ECOR57 are available upon request from the corresponding author A.S.N. The data are not publicly available due to the ongoing research.

## Supplementary Materials

The following are available online at https://www.mdpi.com/article/10.3390/ph15030268/s1, Figure S1: Population dynamics of ECOR57 under SU10 and/or SU57 phage treatments in chemostat microenvironment; Figure S2: Phenotypic diversity among ECOR57 bacteria following phage infection; Figure S3: The genomic relationships between wild-type ECOR57 and ECOR57 with varying morphotypes; Figure S4: One-step growth curves for SU10 used to determine adsorption rates; Figure S5: Chemostat schematics; Table S1: Antibiotic disk-diffusion assay.
