# Supplementary material for: Population Dynamics of a Two Phages–One Host Infection System Using Escherichia coli Strain ECOR57 and Phages vB_EcoP_SU10 and vB_EcoD_SU57
Source: Pharmaceuticals (Basel). 2022 Feb 22;15(3):268. doi: 10.3390/ph15030268 (PMC8953519; doi:10.3390/ph15030268)
Supplement: Supplementary file 1 [file pharmaceuticals-15-00268-s001.zip › pharmaceuticals-1530247-supplementary.pdf]

A

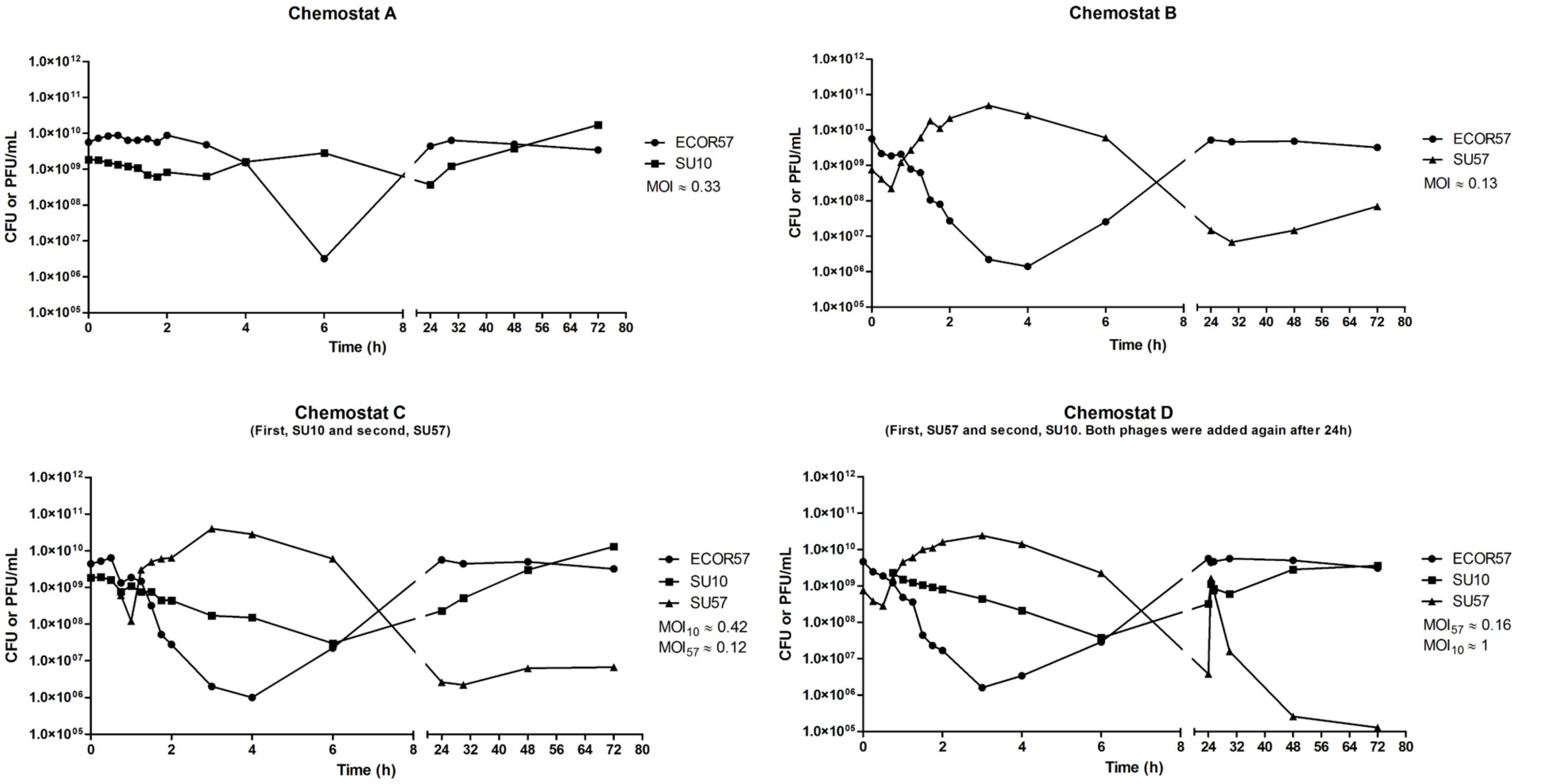
Chemostat A
CFU or PFU/mL
Time (h)
ECOR57
SU10
MOI ≈ 0.33
Chemostat B
CFU or PFU/mL
Time (h)
ECOR57
SU57
MOI ≈ 0.13
Chemostat C
(First, SU10 and second, SU57)
CFU or PFU/mL
Time (h)
ECOR57
SU10
SU57
MOI10 ≈ 0.42
MOI57 ≈ 0.12
Chemostat D
(First, SU57 and second, SU10. Both phages were added again after 24h)
CFU or PFU/mL
Time (h)
ECOR57
SU10
SU57
MOI57 ≈ 0.16
MOI10 ≈ 1

B

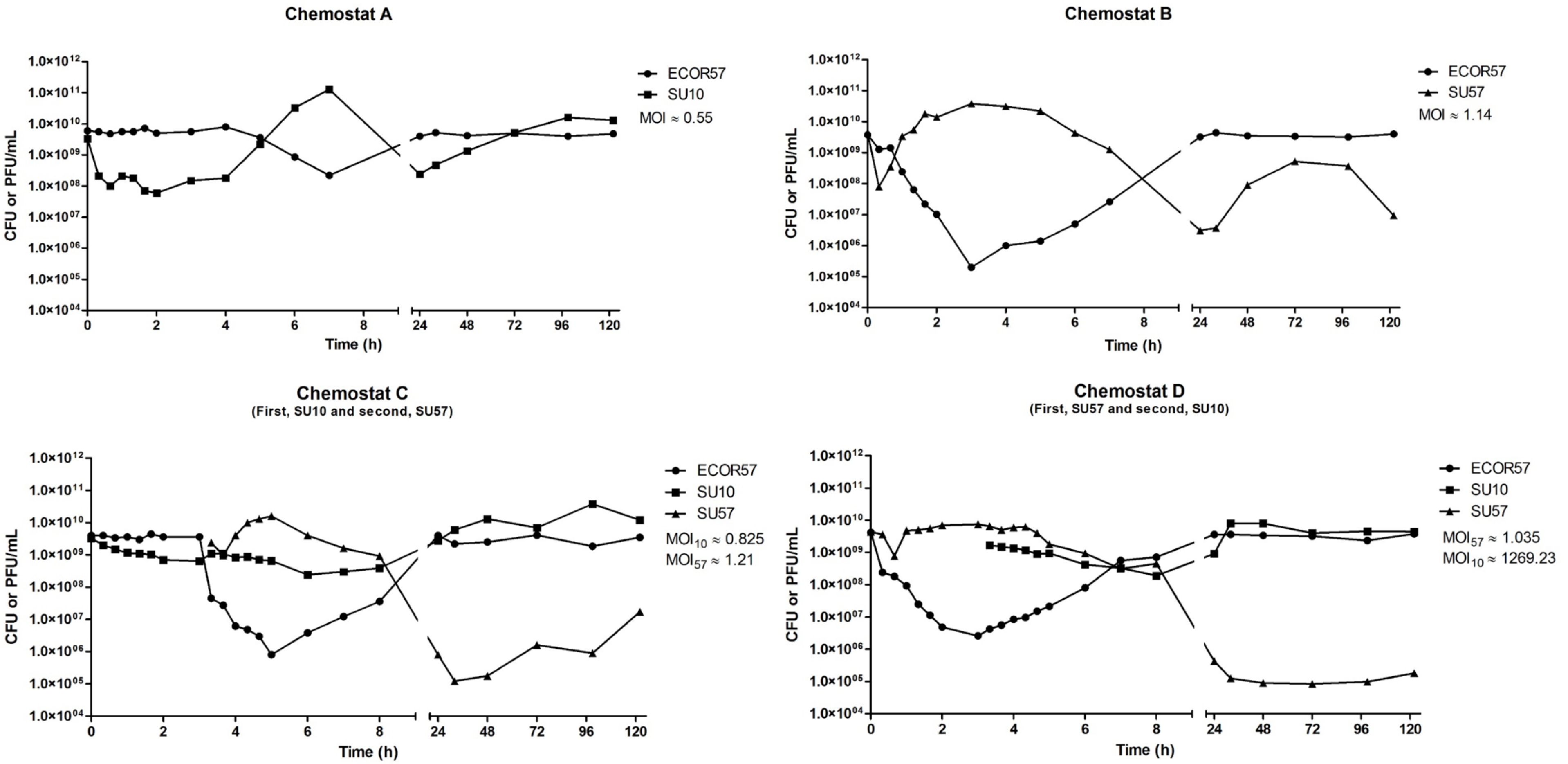
Chemostat A
ECOR57
SU10
MOI ≈ 0.55
CFU or PFU/mL
Time (h)
Chemostat B
ECOR57
SU57
MOI ≈ 1.14
CFU or PFU/mL
Time (h)
Chemostat C
(First, SU10 and second, SU57)
ECOR57
SU10
SU57
MOI10 ≈ 0.825
MOI57 ≈ 1.21
CFU or PFU/mL
Time (h)
Chemostat D
(First, SU57 and second, SU10)
ECOR57
SU10
SU57
MOI57 ≈ 1.035
MOI10 ≈ 1269.23
CFU or PFU/mL
Time (h)

C

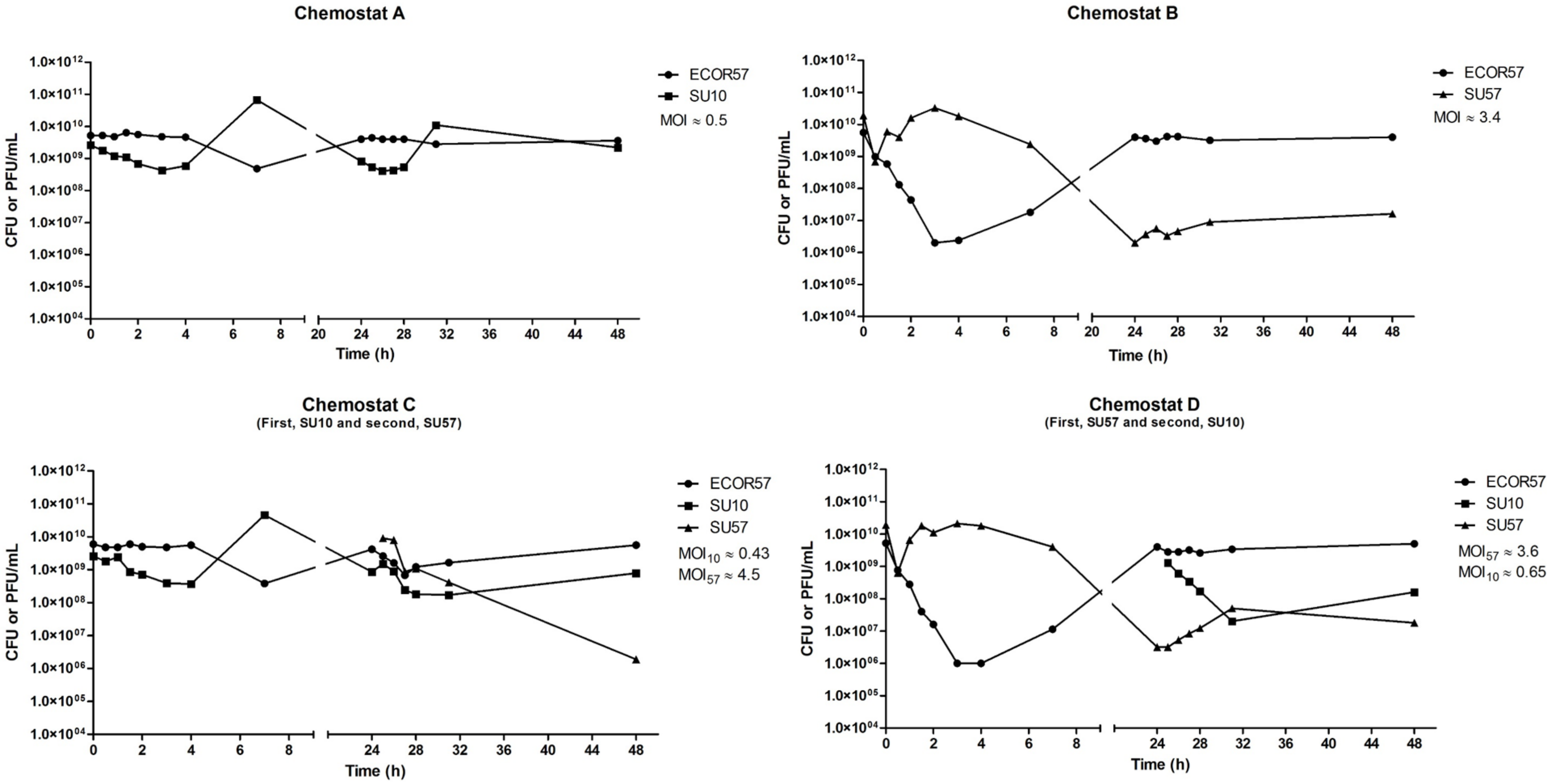
Chemostat A
ECOR57
SU10
MOI ≈ 0.5
CFU or PFU/mL
Time (h)
Chemostat B
ECOR57
SU57
MOI ≈ 3.4
Chemostat C
(First, SU10 and second, SU57)
ECOR57
SU10
SU57
MOI10 ≈ 0.43
MOI57 ≈ 4.5
Chemostat D
(First, SU57 and second, SU10)
ECOR57
SU10
SU57
MOI57 ≈ 3.6
MOI10 ≈ 0.65

Figure S1. Population dynamics of ECOR57 under SU10 and/or SU57 phage treatments in chemostat micro-emvironments. Graphs A, B, and C depict three separate experiments using different MOIs showing the following trends. Infection with SU10 or SU57produced phage-specific population curves. Chemostat A has ECOR57 that has only been infected by SU10. Chemostat B has ECOR57 that has only been infected with SU57. Chemostat C has ECOR57 that was first infected by SU10 then by SU57. Chemostat D has ECOR57 that was first infected by SU57 then by SU10.

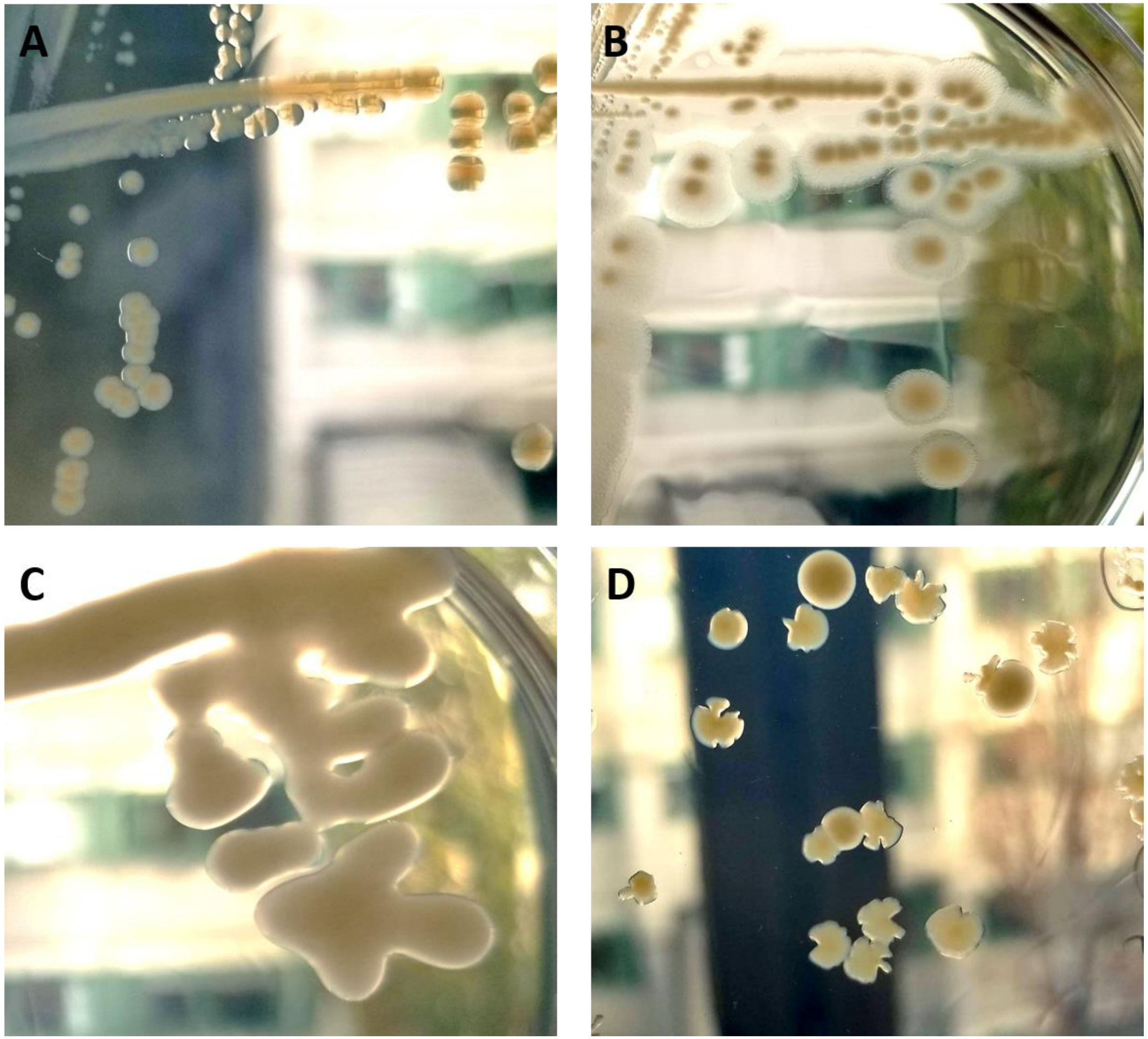

Figure S2. Phenotypic diversity among ECOR57 bacteria following phage infection. A) Normal morphotype B) Edgy morphotype C) Slimy morphotype D) Pointy morphotype

A

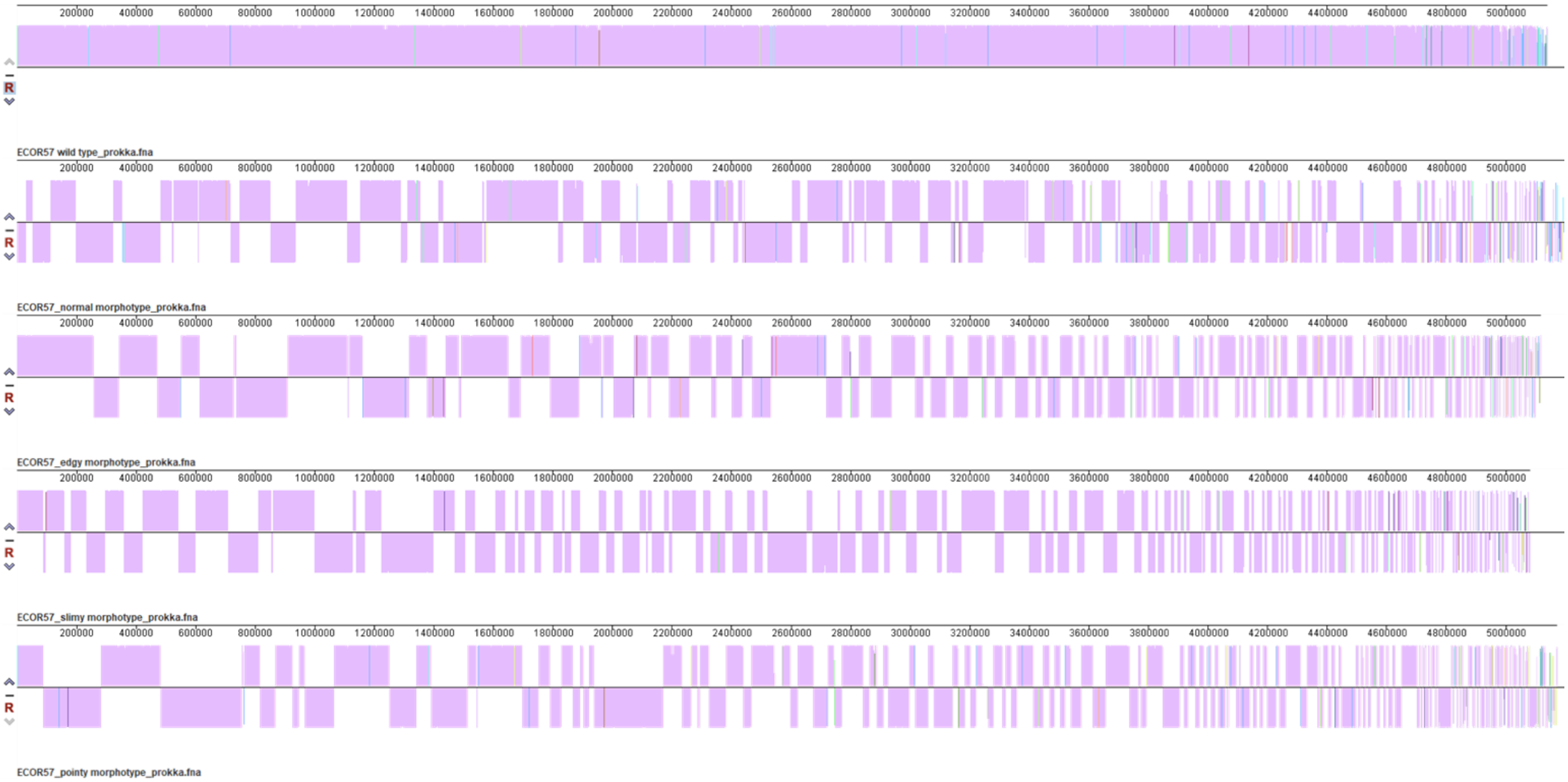
200000 400000 600000 800000 1000000 1200000 1400000 1600000 1800000 2000000 2200000 2400000 2600000 2800000 3000000 3200000 3400000 3600000 3800000 4000000 4200000 4400000 4600000 4800000 5000000
R
ECOR57 wild type_prokka.fna
ECOR57_normal morphotype_prokka.fna
ECOR57_edgy morphotype_prokka.fna
ECOR57_slimy morphotype_prokka.fna
ECOR57_pointy morphotype_prokka.fna

B

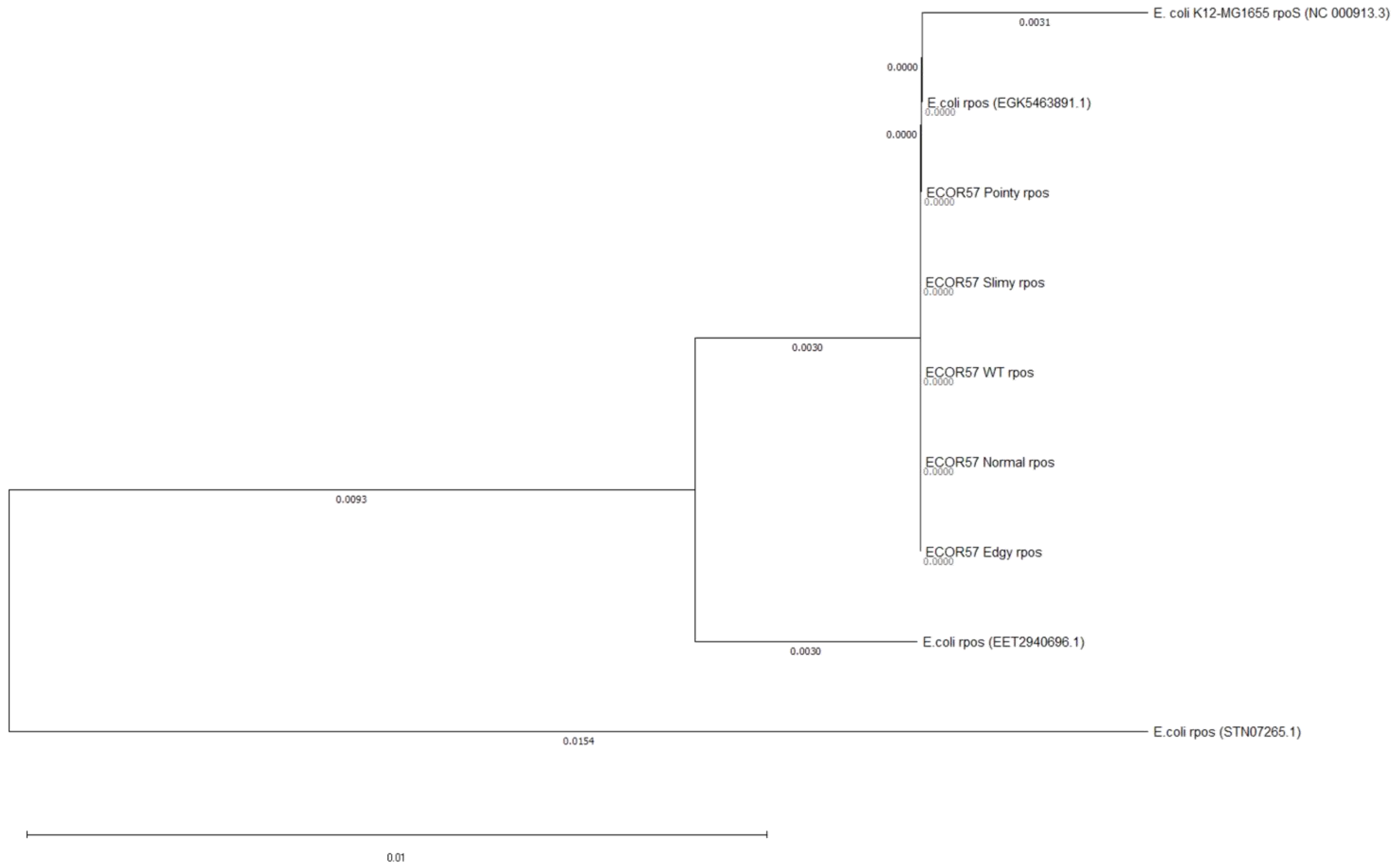

Figure S3. The genomic relationships between wild type ECOR57 and ECO57 with varying morphotypes. A) Multiple genome alignments of wild type ECOR57 and ECOR57 presenting various morphotypes using MAUVE. Bar heights represent the average level of conservation within each region of the sequence. Major conserved regions among all genomes are depicted in mauve coloring. White regions represent fragments that have not been aligned or have sequence specific components. Regions which lie above the center black line are aligned in the forward orientation relative to the wild type ECOR57 reference sequence whereas those below the line indicate regions that align in the reverse complement orientation. B) Neighbor-joining phylogenetic tree representing the relationship between RNA polymerase sigma factor RpoS of ECOR57 in relation to the ECOR57 displaying the various morphotypes, the most commonly used laboratory strain *E. coli* strain K12-MG1655 (NCBI accession number NC_000913.3) and three other *E.coli* strains (NCBI accession numbers EGK5463891.1, EET2940696.1, and STN07265.1).

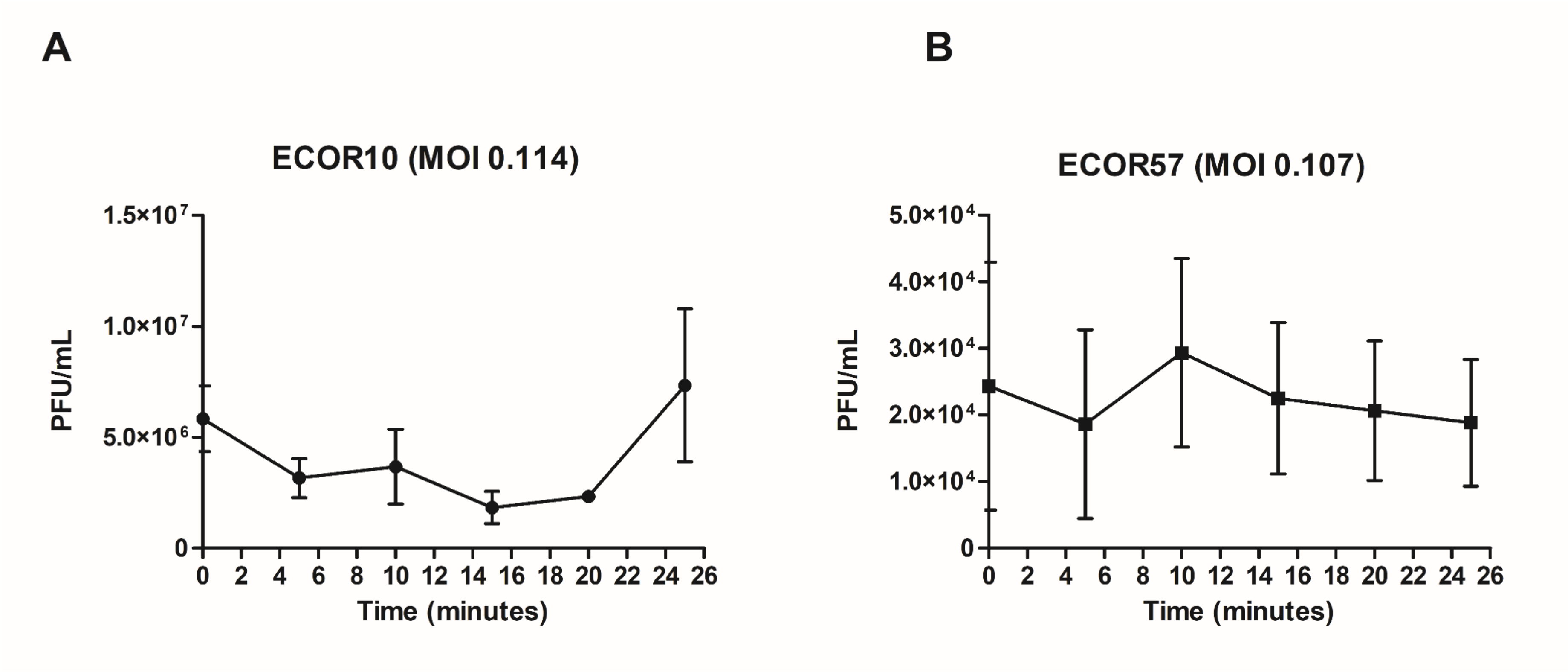

Figure S4. One-step growth curves for SU10 used to determine adsorption rates A) Growth curve of SU10 infecting its host bacteria of ECOR10 at a MOI of 0.114. The adsorption rate five minutes post infection was determined to be 3.10 x 10-9 mL/min. B) Growth curve of SU10 infecting bacteria ECOR57 at a MOI of 0.107. The adsorption rate five minutes post infection was determined to be 1.43 x 10-9 mL/min. Growth curves were obtained using the modified standard protocol (see text for details). Data points represents the mean of three independent experiments and the error bars represent the standard error (SEM) of each run.

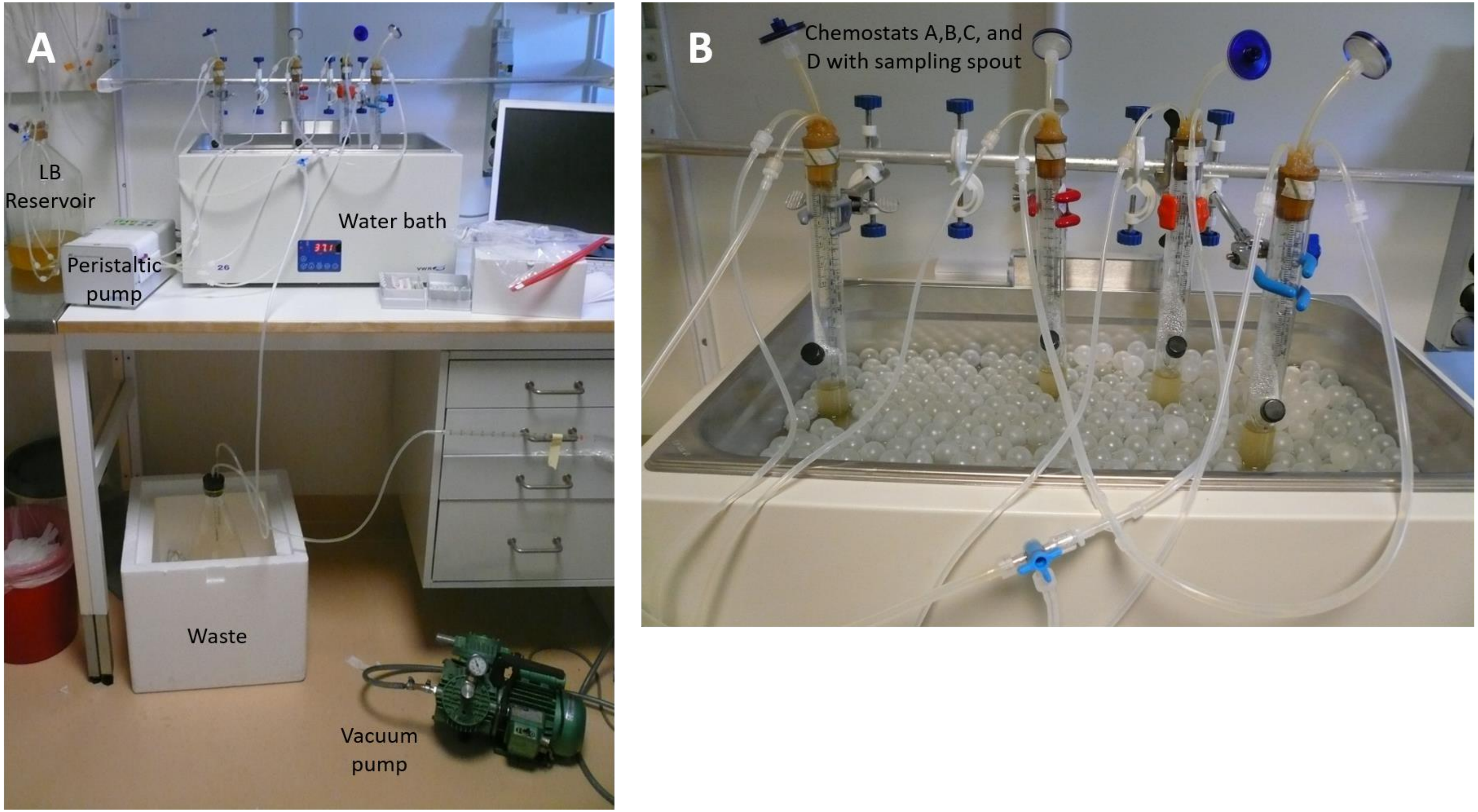

Figure S5. Chemostat schematics. A) Complete chemostat set up with LB reservoir with aeration tube, peristaltic pump, 37°C water bath containing four individual chemostats, waste flask, and vacuum pump. B) Close up of chemostats. Each chemostat was attached to the LB reservoir and waste flask and had an aeration tube fixed with 0.2 µm filter to allow for air flow and contamination avoidance.

Table S1. Antibiotic disk-diffusion assays for varying ECOR57 bacterial morphotypes and those presenting double resistance. Presented are different colonies representing the same phenotype. Zone diameter breakpoints are from the European committee on antimicrobial susceptibility testing (EUCAST) for Enterobacterales. Resistance (R) and susceptibility (S) for the following antibiotics: Ampicillin: ≥ 14 mm is S, < 14 mm is R; Imipenem: ≥ 22 mm is S, < 19mm is R; Ceftazidime: ≥ 22 mm is S, < 19 mm is R. *These samples were grown on different concentrations of ampicillin plates and were determined to be sensitive to ampicillin, albeit less sensitive compared to wild type ECOR57 bacteria.

Sample	Ampicillin		Imipenem		Ceftazidime	
	Zone Diameter (mm)	R/S	Zone Diameter (mm)	R/S	Zone Diameter (mm)	R/S
**ECOR57 WT**	16.0	S	27.0	S	27.5	S
**Normal-3**	20.0	S	30.5	S	29.5	S
**Normal-4**	21.5	S	29.0	S	30.0	S
**Edgy-1**	19.0	S	30.0	S	29.0	S
**Edgy-3**	17.5	S	29.0	S	27.5	S
**Edgy-4**	19.0	S	30.0	S	29.5	S
**Slimy-2**	19.5	S	30.0	S	29.0	S
**Slimy-4**	20.5	S	28.5	S	29.5	S
**Pointy-2**	19.5	S	30.0	S	30.0	S
**Pointy-3**	19.5	S	28.5	S	24.5	S
**DR-1**	12.8	R*	29.6	S	22.8	S
**DR-3**	14.0	S	29.6	S	23.5	S
**DR-4**	13.5	R*	29.8	S	22.8	S
**DR-6**	13.6	R*	29.8	S	22.3	S
**DR-8**	13.1	R*	29.8	S	23.5	S
